# Performance of time-domain and spectral-domain Optical Coherence Tomography for glaucoma screening

**DOI:** 10.1111/j.1755-3768.2010.01977.x

**Published:** 2012-06

**Authors:** Boel Bengtsson, Sabina Andersson, Anders Heijl

**Affiliations:** Department of Clinical Sciences, Ophthalmology in Malmö, Skånes University HospitalMalmö, Sweden

**Keywords:** diagnosis, glaucoma, optical coherence tomography, retinal nerve fibre layer thickness, screening

## Abstract

**Purpose:**

To investigate the measures of validity for selective or population screening of the time-domain Stratus and the spectral-domain Cirrus Optical Coherence Tomography (OCT) imaging techniques in a population-based sample and in clinical glaucoma patients at different stages of glaucoma.

**Patients and methods:**

A random sample of 307 subjects living in two rural areas in southern Sweden, and a random sample of 394 clinical glaucoma patients were selected. A large battery of examinations, including Stratus and Cirrus OCT was performed. OCT retinal nerve fibre layer (RNFL) thickness analyses for average thickness, quadrant and clock-hour sectors were compared with normative significance limits available in the instruments.

**Results:**

The population-based sample included 129 healthy and nine glaucoma subjects, and the sample of clinical glaucoma patients included 138 patients. Specificity and positive predictive values were generally better with Stratus than for Cirrus, and sensitivity was generally better with Cirrus. With the average RNFL thickness parameter, Stratus reached 100% specificity and a positive predictive value of 100% and 68% sensitivity in the whole group of the clinical glaucoma patients, but sensitivity was only 28% among the earliest stage glaucoma patients. Sensitivity increased considerably when relying on the quadrant sector parameter, while specificity decreased only marginally.

**Conclusion:**

Stratus, with high specificity and positive predictive values, seemed to be best of choice for screening purposes, while Cirrus, with high sensitivity, was the better choice for early detection.

## Introduction

In the western world, approximately 50% of all glaucoma patients are undetected (Bengtsson 1981; Sommer et al. 1991; Dielemans et al. 1994; Leske et al. 2001). We have taken interest in investigating validity of retinal nerve fibre layer (RNFL) measurements by Optical Coherence Tomography (OCT) imaging techniques for screening of glaucoma. The use of RNFL measurements for the diagnosis of glaucoma has increased considerably since the development of OCT imaging techniques (Huang et al. 1991; Schuman et al. 1995). Time-domain OCT is a two-dimensional imaging method based on low-coherence interferometry that noninvasively produces cross-sectional retinal images. This technique makes it possible to quantify the thickness of the retina and its different layers. The first generation of time-domain OCT instruments became available in the 1990s, and in the intervening years OCT has evolved towards higher spatial resolution and faster scan speeds. For detection of glaucoma, circular scans around the optic nerve head provide RNFL thickness measurements of the peripapillary area.

Recently, new OCT instruments using spectral-domain technology have been developed. Compared to time-domain, spectral-domain OCT provides improved axial resolution, and increases in scanning speed of more than a factor of 50. For glaucoma diagnosis, the spectral-domain Cirrus OCT (Carl Zeiss Meditec, Dublin, CA, USA) extracts peripapillary scans from a high-resolution three-dimensional scan cube covering a 6 × 6 × 2 mm volume centred on the optic disc to form a scan ring comparable to that produced by the Stratus OCT. Analysis tools referring to normative significance limits for RNFL thickness are available both for Stratus and for Cirrus.

A number of studies reporting the diagnostic accuracy of Stratus have been published (Chen & Huang 2005; Jeoung et al. 2005; Budenz et al. 2005; DeLeón-Ortega et al. 2006; Manassakorn et al. 2006; Sihota et al. 2006; Hougaard et al. 2007; Lu et al. 2008; Yoo & Park 2009 e-pub, Sehi et al. 2009; Vessani et al. 2009), generally with higher specificities, often round 90%, than sensitivities, typically ranging from 70% to 80%. A few more recent studies comparing Stratus and Cirrus reported similar or slightly better diagnostic accuracy with Cirrus than with Stratus (Leung et al. 2009; Sung et al. 2009; Park et al. 2009; Chang et al. 2009; Knight et al. 2009; Vizzeri et al. 2009; Jeoung & Park 2010; Moreno-Montañés et al. 2010). These studies included glaucoma patients under clinical care. The normal subjects were often recruited among patients with apparently healthy eyes, relatives to patients and hospital staff.

While specificity and sensitivity values depend upon the normal and diseased populations being evaluated, they may be regarded within those limitations as stable properties of a diagnostic test. Predictive values are additionally affected by sample disease prevalence. When considering a method's suitability for population screening, or perhaps for selective screening, any evaluation should be performed in a population similar to the target group. Many diagnostic modalities have been validated in groups that have disease prevalences that are not at all representative of the general population e.g. in clinical glaucoma patients. Predictive values calculated on the basis of such samples would then be artificially high compared to what might be found in population studies.

It is important for a screening method not to miss subjects with advanced disease. A method failing to identify subjects at advanced stages probably should not be used, at least not for population screening. Detection of advanced disease is usually easy with 100% sensitivity for most diagnostic methods, also crude ones, but it does not seem to be true for all techniques (Nguyen et al. 2002; Reus & Lemij 2004). Presuming that most subjects identified in a population-based sample have early disease makes it necessary to evaluate sensitivity in clinical glaucoma patients at different stages of glaucomatous disease.

The aim of our study was to investigate the diagnostic performance in terms of sensitivity, specificity and predictive values of time-domain Stratus and spectral-domain Cirrus OCT for use in population or selective glaucoma screening.

## Methods

### Subjects – recruitment

#### Population-based sample

Out of 4718 subjects over 50 years of age living in two primary medical care districts in southern Sweden, a random sample of 307 individuals was selected and invited by mail to come to their primary care centre for a comprehensive ophthalmic examination carried out for scientific purposes. All invited subjects received a phone call explaining the purpose of the study. Reasons for not wanting or being able to participate were registered.

#### Clinical glaucoma patients

A random sample of 394 patients having diagnoses of either primary open angle glaucoma or pseudo-exfoliation glaucoma was selected from the clinical directory of visits for the previous twelve months at the department of Ophthalmology, Malmö University Hospital, Sweden. Records were missing for 24 of the selected patients. All other records were prescreened to assure that those invited were free of co-morbidities that were likely to confound our analysis or were deceased. Five had moved out of the region, and 29 had secondary or angle closure or suspect glaucoma. Eighty patients declined to come, and two did not show up to the scheduled visits. A total of 164 patients participated. Nineteen patients, who were examined during the first days of the data collection, were excluded from the study because the early Cirrus OCT software did not include the peripapillar scan protocol. Another six patients were excluded, of whom three were not able to complete the examinations, and another three because of software problem with Cirrus.

For eligibility, the optic disc had to show glaucomatous changes assessed from a conventional photograph and/or described in the patient record. To be included in the analysis, glaucomatous disc finding had to be confirmed at the study visit. Other diagnostic findings such as visual field status, intraocular pressure or results from imaging devices were not considered before inclusion.

All population and clinical participants were informed about the purpose of the examination, and all included gave informed consent. The tenets of the Declaration of Helsinki were followed, and the Regional Ethical Review Board in Lund, Sweden approved the study.

### Examinations

All subjects and patients were asked about their medical history and medications and underwent the following examinations of both eyes:

1. Autorefraction and determination of visual acuity. Manual refraction and test of visual acuity was performed only when autorefractor-based visual acuity was <0.8.
2. Measurement of intraocular pressure using a Goldmann applanation tonometer.
3. Fundus examination by biomicroscopy through a dilated pupil.

Among subjects in the population-based sample, one eye was randomly selected for further diagnostic evaluation. Subjects found to have nonglaucomatous disease known to affect the optic disc, the RNFL or the visual field were excluded from the analysis. In clinical patients having bilateral glaucoma, the eye with the better perimetric mean deviation (MD) value was selected. Selected eyes were further examined with the following:

1. Scanning Laser Polarimetry using the GDx VCC instrument with Revision 5.5.0 software in the screening mode (Carl Zeiss Meditec).
2. Frequency Doubling Technology (FDT) perimetry (Carl Zeiss Meditec) screening program C20-1.
3. Standard Automated Perimetry (SAP) using the 24-2 SITA Standard program of the Humphrey perimeter (Carl Zeiss Meditec).
4. Time-domain Stratus OCT examination (Carl Zeiss Meditec) using the Fast RNFL thickness protocol of three 3.4-mm-diameter peripapillary circular scans each of 256 scans measuring points continuously captured through a dilated pupil.
5. Spectral-domain Cirrus OCT examination (Software Version 3.0, Carl Zeiss Meditec) using a scan cube measuring a 6 × 6 mm area with 200 B-scans and 200 A-scans per B-scan, captured through a dilated pupil. From this data cube, a 3.4-mm-diameter circle is automatically extracted by the instrument's analysis software to form the basis for the RNFL thickness measurement.

All examinations except OCT were performed by one of the authors (SA). The same experienced ophthalmic photographer performed all OCT examinations.

### OCT parameters

Analysis tools referring to normal RNFL thickness database are available in the software of both instruments for average RNFL thickness, mean quadrant thickness, and the mean thickness in each of 12 peripapillary clock-hour sectors. RNFL parameters falling outside the lower 5th or 1st percentile significance limits of the instruments’ respective normative databases are highlighted as significantly depressed.

## Analyses

In the population-based sample, we calculated sensitivity, specificity and predictive values for average, quadrant and clock-hour RNFL thickness, using the p < 5% and p < 1% significance limits as cut-off values for positive findings. For quadrants and clock-hours, areas under the receiver operating characteristic curves (AROC) were calculated using spss version 16.0 (SPSS Inc., Chicago, IL, USA).

Sensitivity values were calculated for the complete group of clinical glaucoma patients, as well as for subgroups at different stages of disease. Patients were divided into four groups by the perimetric mean deviation (MD) as follows:

early glaucoma: MD > −6 dB
moderate glaucoma: −6 dB ≥ MD > −12
severe glaucoma: −12 dB ≥ MD > −18 dB
advanced glaucoma: MD ≤ −18 dB

## Results

### Population-based subjects

One hundred and seventy subjects, 55%, of all invited in the population-based random sample responded to the invitation and received the comprehensive ophthalmic examination. The most common reason for not participating was inability or unwillingness to attend the examination. Five subjects of those declining were already under ophthalmological care; one had age-related macular degeneration, one retinitis pigmentosa, one diabetic retinopathy, one had glaucoma and another had been identified as a glaucoma suspect.

Of those participating, three subjects were discovered to already have prior diagnoses of glaucoma. Another 15 subjects were found in our examination to have abnormal discs as assessed by fundus biomicroscopy or results that were outside normal limits on one or more diagnostic tests. These were all scheduled for further examinations at the department of Opthalmology in Malmö. Two of the 15 subjects were lost to follow-up; one moved out of the country, and the other had a serious systemic disease making it impossible to continue in the study. All follow-up examinations were performed by one of the authors (AH). Six of the 15 subjects were given a diagnosis of glaucoma on the basis of follow-up examination. All six had reproducible visual field defects and Glaucoma Hemifield Test findings that were outside normal limits. Five of the six had glaucomatous discs, e.g. notching or cupping reaching the disc margin, and one of the five had a disc haemorrhage and exfoliations. The sixth subject had suspicious disc findings that were consistent with the visual field test results. Further, two subjects had suspect glaucoma and, in the absence of a clear-cut findings/classification, were not included in the analysis. Seven of the 15 were considered normal and included in the analysis as healthy subjects, resulting in a total of 129 healthy subjects.

Among the population subjects examined, six were excluded from the analysis; three had visual field defects consistent with neurological disease, two had age-related macular disease, and one had optic disc drusen.

**Fig. 1:**
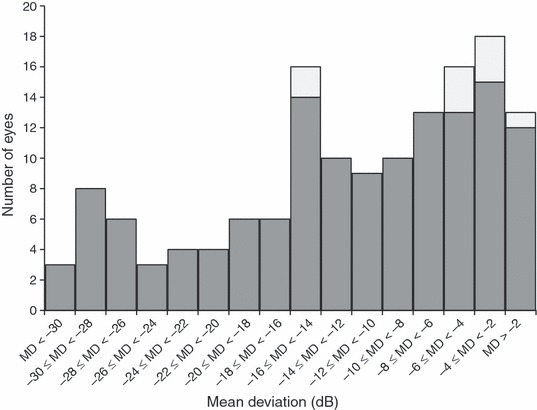
Mean Deviation (MD) values of visual fields assessed in clinical glaucoma patients (dark bars) and in the nine subjects in the population-based sample who were found to be glaucomatous (white bars). Seven of the latter nine patients had mild visual field defects with MD values better than −6 dB, and two subjects, both previously diagnosed, had MD values of approximately −15 dB.

Thus, in the analysis of the population-based sample, we identified nine subjects with glaucoma, three previously diagnosed and six newly detected giving a glaucoma prevalence of 6.5%. In the nine subjects with glaucoma, the perimetric mean deviation values (MD) ranged from −15.87 to +0.07 dB (median −4.58 dB), Fig. 1.

The mean age of the nine glaucoma subjects was 72 years, ranging from 61 to 83, while the mean age of the 129 healthy subjects was 64 years, ranging from 48 to 81. Three of the nine glaucoma subjects (33%) and 55% of the healthy subjects were women.

OCT scans were generally of high quality; all but one had signal strength above the minimum level recommended by the manufacturer, i.e. ≥5 for Cirrus in version 3.0 and ≥7 for Stratus in version 4.0.4. Average RNFL was generally thicker with Stratus than with Cirrus, 101 and 88 μm, respectively, in the healthy subjects.

### Clinical glaucoma patients

Findings from one eye from each of 138 glaucoma patients, 59% women, with a mean age of 71 years, were included in the random sample of clinical patients. Perimetric MD values ranged from −31.54 to +0.28 dB, with a median of −11.08 dB, Fig. 1.

Also in the clinical glaucoma patients, OCT scans were generally of high quality, 93% had a signal strength ≥5 with Cirrus and 93%≥7 with Stratus. Average RNFL thickness was 61 μm with Cirrus and 63 μm with Stratus, a smaller difference than that obtained in the healthy subjects.

### Sensitivity, specificity and predictive values

In the sample of clinical glaucoma patients, sensitivity was better for Cirrus than for Stratus, particularly at early stages of glaucoma, Table 1. The difference between Cirrus and Stratus decreased with increasing severity of disease and was similar or identical at severe and advanced stages where both instruments had 100% sensitivity with all quadrant and clock-hour sector analyses. The difference in sensitivity between Cirrus and Stratus was larger with the p < 5% as cut-off than when using p < 1%.

**Table 1:** Diagnostic sensitivity of Optical Coherence Tomography Retinal Nerve Fibre Layer thickness (RNFLT) analyses in a random sample of clinical glaucoma patients

		Sensitivity for average RNFLT		Sensitivity for quadrant sector RNFLT (≥1 quadrant)		Sensitivity for clock-hour sector RNFLT (≥1 clock hour)	
				
		Cirrus	Stratus	Cirrus	Stratus	Cirrus	Stratus
Overall (*n* = 138)	Cut-off p < 5%	0.90 (0.83–0.94)	0.78 (0.70–0.85)	0.96 (0.90–0.98)	0.93 (0.87–0.96)	0.94 (0.89–0.97)	0.95 (0.89–0.98)
	Cut-off p < 1%	0.73 (0.65–0.80)	0.68 (0.60–0.76)	0.91 (0.85–0.95)	0.83 (0.76–0.89)	0.93 (0.87–0.96)	0.93 (0.87–0.96)
MD > −6 dB (*n* = 40)	Cut-off p < 5%	0.78 (0.61–0.89)	0.45 (0.30–0.61)	0.90 (0.75–0.97)	0.80 (0.64–0.90)	0.88 (0.72–0.95)	0.85 (0.69–0.94)
	Cut-off p < 1%	0.38 (0.23–0.54)	0.28 (0.15–0.44)	0.78 (0.61–0.89)	0.60 (0.43–0.75)	0.80 (0.64–0.90)	0.60 (0.43–0.75)
−6 dB ≥ MD > −12 dB (*n* = 34)	Cut-off p < 5%	0.88 (0.72–0.96)	0.82 (0.65–0.93)	0.94 (0.79–0.99)	0.94 (0.79–0.99)	0.97 (0.83–1.00)	0.97 (0.83–1.00)
	Cut-off p < 1%	0.76 (0.58–0.89)	0.74 (0.55–0.86)	0.91 (0.75–0.98)	0.85 (0.68–0.94)	0.94 (0.79–0.99)	0.94 (0.79–0.99)
−12 dB ≥ MD > −18 dB (*n* = 30)	Cut-off p < 5%	0.97 (0.81–1.00)	0.97 (0.81–1.00)	1.00 (0.86–1.00)	1.00 (0.86–1.00)	1.00 (0.86–1.00)	1.00 (0.86–1.00)
	Cut-off p < 1%	0.93 (0.76–0.99)	0.90 (0.72–0.97)	1.00 (0.86–1.00)	1.00 (0.86–1.00)	1.00 (0.86–1.00)	0.97 (0.81–1.00)
MD ≤ −18 dB (*n* = 34)	Cut-off p < 5%	1.00 (0.87–1.00)	1.00 (0.87–1.00)	1.00 (0.87–1.00)	1.00 (0.87–1.00)	1.00 (0.87–1.00)	1.00 (0.87–1.00)

The best AROC, 0.99, was obtained with Cirrus quadrant sector parameter with a cut-off level for pathology at p < 1%. This parameter yielded a specificity of 96%, positive predictive value of 64% and sensitivity of 100% in the population sample (Table 2). The sensitivity was 91% in the whole group of the clinical glaucoma patients and of 78% in those 40 clinical glaucoma patients with early glaucoma (Table 1).

**Table 2:** Diagnostic performance of Optical Coherence Tomography Retinal Nerve Fibre Layer thickness (RNFLT) analyses in a population-based random sample

		Average RNFLT		Quadrant sector RNFLT (≥1 quadrant)		Clock hour sector RNFLT (≥1 clock hour)	
				
		Cirrus	Stratus	Cirrus	Stratus	Cirrus	Stratus
AROC (95% CI)	Cut-off p < 5%			0.96* (0.93–1.00)	0.93* (0.80–1.00)	0.94† (0.88–1.00)	0.97† (0.93–1.00)
	Cut-off p < 1%			0.99* (0.97–1.00)	0.81* (0.60–1.00)	0.97† (0.94–1.00)	0.82† (0.63–1.00)
Sensitivity (95% CI)	Cut-off p < 5%	0.89 (0.68–1.00)	0.78 (0.51–1.00)	1.00 (1.00–1.00)	0.89 (0.68–1.00)	1.00 (1.00–1.00)	1.00 (1.00–1.00)
	Cut-off p < 1%	0.67 (0.36–0.98)	0.67 (0.36–0.98)	1.00 (1.00–1.00)	0.67 (0.36–0.98)	1.00 (1.00–1.00)	0.67 (0.36–0.98)
Specificity (95% CI)	Cut-off p < 5%	0.95 (0.92–0.99)	0.99 (0.98–1.00)	0.81 (0.74–0.88)	0.93 (0.89–0.97)	0.65 (0.57–0.73)	0.81 (0.74–0.87)
	Cut-off p < 1%	0.98 (0.95–1.00)	1.00 (1.00–1.00)	0.96 (0.93–1.00)	0.98 (0.95–1.00)	0.91 (0.86–0.96)	0.97 (0.94–1.00)
Positive predictive value (95% CI)	Cut-off p < 5%	0.57 (0.31–0.83)	0.88 (0.65–1.00)	0.27 (0.12–0.41)	0.47 (0.23–0.71)	0.17 (0.07–0.27)	0.27 (0.12–0.41)
	Cut-off p < 1%	0.67 (0.36–0.98)	1.00 (1.00–1.00)	0.64 (0.39–0.89)	0.67 (0.36–0.98)	0.43 (0.22–0.64)	0.60 (0.30–0.90)
Negative predictive value (95% CI)	Cut-off p < 5%	0.99 (0.98–1.00)	0.99 (0.96–1.00)	1.00 (1.00–1.00)	0.99 (0.98–1.00)	1.00 (1.00–1.00)	1.00 (1.00–1.00)
	Cut-off p < 1%	0.98 (0.95–1.00)	0.98 (0.95–1.00)	1.00 (1.00–1.00)	0.98 (0.95–1.00)	1.00 (1.00–1.00)	0.98 (0.95–1.00)

AROC, area under the receiver operating characteristic curve.

95% CI, confidence intervals at the 95% significance level in parenthesis.

* AROCs for all quadrant sectors.

† AROCs for all clock-hour sectors.

Specificity and positive predictive values were good with Cirrus, and often excellent with Stratus, Table 2. The average thickness parameter, using p < 1% as the cut-off level for pathology, yielded specificity and positive predicted value of 100% (Table 2). The sensitivity for this parameter was 67% based on the nine glaucoma subjects in the population sample and 68% among the 138 clinical glaucoma patients, but only 28% in those 40 clinical patients having early glaucoma (Table 1). When defining pathology as requiring at least one ‘quadrant sector’ below the p < 1%, the sensitivity for Stratus increased to 60% in the 40 clinical patients with early glaucoma, to 83% in the overall sample of clinical glaucoma patients (Table 1) and remained at 67% in the glaucoma subjects in the population sample, while specificity decreased slightly to 98% and the positive predictive value to 67% (Table 2).

## Discussion

The performances of both instruments were high in our population-based sample, often with AROCs well above 0.90 (Table 2). We chose to investigate the diagnostic performance of RNFL thickness measurements assessed by the circular scan around the optic nerve head because both time-domain Stratus and the spectral-domain Cirrus OCT provide analysis tools in the form of normative limits for that type of scan. Cirrus also provides an en face view of RNFL thickness and deviations from normal using the cube of OCT data, but we did not include this feature in our analyses.

Generally, specificity was higher for Stratus than for Cirrus. For all parameters and both cut-off levels, p < 5% and p < 1%, positive predicted values were better for Stratus than for Cirrus. Correspondingly, sensitivity was higher for Cirrus, which also had the best AROC of 0.99.

In the population-based sample, the Stratus ‘average thickness parameter’ using p < 1% as cut-off resulted in specificity and positive predictive value of 100% each and 67% sensitivity. The positive predictive value is remarkably good considering its strong correlation to the prevalence of the disease. At 6.5%, the prevalence of glaucoma found in our study was similar to the 6% value reported in meta-analysis by Rudnicka et al. (2006) in a white population older than 70 years. Thus, despite our relatively small population sample size the prevalence seems representative for a white population.

Specificity was often better than expected considering that we used the manufacturer's normative limits at the p < 5% and p < 1% levels as cut-off for positive findings. This might be explained by the demographics of our population-based sample including only subjects from a suburban area in southern Sweden with an almost 100% white population, and thereby avoiding possible variability in RNFL thickness induced by subjects of different ethnicities. Another possible explanation is that the same photographer, very experienced in acquisition of OCT data, performed all examinations.

The important measures for a screening test, specificity and positive predictive value were estimated among the 129 healthy and nine glaucoma subjects identified in the population sample. Of course, it would have been desirable to find more glaucoma patients in our population survey, but with a response rate of 55%, and a glaucoma prevalence of 6.5%, as in our study, we would have had to invite 2800 subjects, and to examine more than 1500 to find 100 glaucoma patients. Therefore, we included clinical glaucoma patients to assess sensitivity.

The high specificity and positive predictive values suggest that the Stratus average RNFL thickness parameter, with a cut-off at the p < 1% level, could be the best choice for population screening purposes if we accept not being able to detect many of the earliest cases. In a previous study Hougaard et al. (2008) reported that very localized RNFL defects that were visible in images may be missed by the Stratus analysis package (Carl Zeiss Meditec, Dublin, CA, USA). Sensitivity increased considerably when using the quadrant and the clock-hour sector analyses of RNFL thickness. The sector analyses yielded 100% sensitivity with both Cirrus and Stratus in patients with severe and advanced glaucomatous visual field loss, i.e. MD worse than −12 dB (Table 1). The quadrant sector analysis using Stratus with a cut-off at p < 1% yielded high specificity (98%) and positive predictive value (75%) (Table 2), and the sensitivity increased from 28% to 60% in the group of clinical patients with earliest glaucoma (Table 2). Thus, it seems as OCT Stratus sector analysis with a cut-off at p < 1% could serve well as screening instrument.

Tests used in population screenings should be simple, rapid, inexpensive and safe. OCT examinations are rapid, about 5 min with Stratus and 3–4 min with Cirrus. OCT examinations are also safe. The technologies are not simple enough for untrained technicians to carry out the examinations, and the instruments are not inexpensive. Nevertheless, OCT seems to have a potential as a screening tool for glaucoma in an affluent society, but it would be desirable to have our results confirmed in other population-based studies before starting large-scale population screening.

Some of the differences between the results obtained with the two instruments might be because of differences between the two normative databases used to generate normal limits. Thus, the Cirrus database includes a much higher percentage of patients of Asian ethnicity (Cirrus 4.9 User Manual; Carl Zeiss Meditec). Because average RNFL thickness is probably thicker in Asians, one would expect that this has resulted in somewhat higher RNFL values than if the database included only small number of Asian subjects like for Stratus (Budenz et al. 2007).

With most OCT parameters, Stratus yielded better specificity and positive predictive values, both important properties of a screening test, than Cirrus. However, in settings giving priority to early detection and high sensitivity, e.g. in clinical settings, Cirrus is the better choice because sensitivity with Cirrus generally was higher than for Stratus.
